# Supplementary material for: HIVprotI: an integrated web based platform for prediction and design of HIV proteins inhibitors
Source: J Cheminform. 2018 Mar 9;10:12. doi: 10.1186/s13321-018-0266-y (PMC5845081; doi:10.1186/s13321-018-0266-y)
Supplement: Supplementary file 2 — Additional file 2. Source code of HIVProtI web server. [file 13321_2018_266_MOESM2_ESM.zip › HIVprotI_Source-code/hivprotI/weka-3-6-6/doc/weka/classifiers/package-summary.html]

weka.classifiers


---


|  |  |  |
| --- | --- | --- |
| **Overview** \| **Package** \| Class \| **Tree** \| **Deprecated** \| **Index** \| **Help** \| **Weka's home** | |  |
| **PREV PACKAGE**   **NEXT PACKAGE** | **FRAMES**    **NO FRAMES**     **All Classes** |  |


---

## Package weka.classifiers

| **Interface Summary** | |
| --- | --- |
| **IntervalEstimator** | Interface for classifiers that can output confidence intervals |
| **IterativeClassifier** | Interface for classifiers that can induce models of growing complexity one step at a time. |
| **Sourcable** | Interface for classifiers that can be converted to Java source. |
| **UpdateableClassifier** | Interface to incremental classification models that can learn using one instance at a time. |

| **Class Summary** | |
| --- | --- |
| **BVDecompose** | Class for performing a Bias-Variance decomposition on any classifier using the method specified in:    Ron Kohavi, David H. |
| **BVDecomposeSegCVSub** | This class performs Bias-Variance decomposion on any classifier using the sub-sampled cross-validation procedure as specified in (1).  The Kohavi and Wolpert definition of bias and variance is specified in (2).  The Webb definition of bias and variance is specified in (3).    Geoffrey I. |
| **CheckClassifier** | Class for examining the capabilities and finding problems with classifiers. |
| **CheckSource** | A simple class for checking the source generated from Classifiers implementing the `weka.classifiers.Sourcable` interface. |
| **Classifier** | Abstract classifier. |
| **CostMatrix** | Class for storing and manipulating a misclassification cost matrix. |
| **Evaluation** | Class for evaluating machine learning models. |
| **IteratedSingleClassifierEnhancer** | Abstract utility class for handling settings common to meta classifiers that build an ensemble from a single base learner. |
| **MultipleClassifiersCombiner** | Abstract utility class for handling settings common to meta classifiers that build an ensemble from multiple classifiers. |
| **RandomizableClassifier** | Abstract utility class for handling settings common to randomizable classifiers. |
| **RandomizableIteratedSingleClassifierEnhancer** | Abstract utility class for handling settings common to randomizable meta classifiers that build an ensemble from a single base learner. |
| **RandomizableMultipleClassifiersCombiner** | Abstract utility class for handling settings common to randomizable meta classifiers that build an ensemble from multiple classifiers based on a given random number seed. |
| **RandomizableSingleClassifierEnhancer** | Abstract utility class for handling settings common to randomizable meta classifiers that build an ensemble from a single base learner. |
| **SingleClassifierEnhancer** | Abstract utility class for handling settings common to meta classifiers that use a single base learner. |

---


|  |  |  |
| --- | --- | --- |
| **Overview** \| **Package** \| Class \| **Tree** \| **Deprecated** \| **Index** \| **Help** \| **Weka's home** | |  |
| **PREV PACKAGE**   **NEXT PACKAGE** | **FRAMES**    **NO FRAMES**     **All Classes** |  |


---
